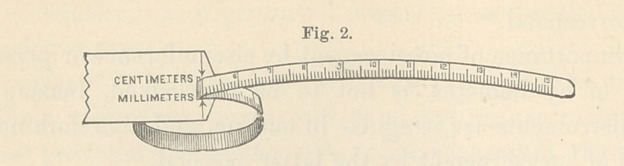# The Perimetric Dimension System; a General System of Measurement for Urethral, Uterine, Rectal and Other Instruments; and an Adaptable Metric Gauge*Exhibited to the Philadelphia County Medical Society, June 25th, 1879.

**Published:** 1879-12

**Authors:** Charles Hermon Thomas

**Affiliations:** Fellow of the College of Physicians, etc. of Philadelphia, Pa.


### THE PERIMETRIC DIMENSION SYSTEM; A GENERAL SYSTEM OF MEASUREMENT FOR URETHRAL, UTERINE, RECTAL AND OTHER INSTRUMENTS: AND AN ADAPTABLE METRIC GAUGE.* By Charles Hermon Thomas, M.D., Fellow of the College of Physicians, etc. of Philadelphia, Pa.

Three scales for grading and numbering urethral instruments are now in use in the United States, each scale having distinct characteristics. The differences between them are radical and material, and they are not accurately interconvertible. Of these conflicting standards the universally known French scale is doubtless usually preferred, and indications are not wanting which point to its general adoption. The English scale, formerly almost exclusively used, is purely arbitrary in character; has proved inaccurate in practice; is inconveniently limited in its range of sizes, and is rapidly falling into disuse; while the American scale, somewhat recently introduced — though undoubtedly an improvement on the English — is at least lacking in simplicity, and its claim to supplant the French has not been justified.

According to the French scale, each size in a set of catheters or bougies is derived from, and identical with the number of millimeters in circumference which such instrument actually measures — an arrangement at once rational and simple. Thus, while No. 1 is 1 M. M. in circumference, No. 2 is 2 M. M., No. 3, 3 M. M, and so on uniformly throughout.

The American scale, though like the French founded on the metric system, has for its gradations half millimeters in

* Exhibited to the Philadelphia County Medical Society, June 25th, 1879.

diameter, instead of whole millimeters in circumference. Its numbers, however, are consecutive in units, and therefore correspond neither with the figures which represent diameters nor circumferences. Pratically it differs from the latter in that it does away with one in every three of the French sizes — a somewhat questionable improvement, though the only merit claimed for it; and in doing this a new and arbitrary series of numbers is introduced — a serious disadvantage. Thus, while No. 1 is 1 M. M. in diameter, No. 2 is 1.5 M. M., No. 3 is 2 M. M., and so on, with a widening disparity till No. 20 is reached, which measures 10.5 M. M. by the same method.

It will readily be conceded that the demand among those engaged in general scientific work for unity of standard in measures of length, capacity and weight, which has resulted in the wide-spread adoption of the metric system, has a practical basis. Nor will it be questioned that the various branches of the science of medicine have need of the improved methods and means of observation and experiment which have become common to allied sciences. In the subdepartments of urethral, gynæcic and rectal surgery especially, there is urgent need for the establishment of a common standard of measurement and record of the dimensions of the instrument employed; and — no less important — by means of these of the calibre of the passages to which they relate.

A general system suited to this wide range of applications, is practicable, and an undoubted necessity — a system combining the requisites of simplicity, definiteness and convenience of use, together with universal scientific intelligibility. The attainment of this end requires

*Fig. 1.*

simply the abandonment of all conventional numbers, whether arbitrary or systematic, as indicative of size, and the adoption of actual circumferential or perimetric dimensions, expressed in terms of the metric unit.

This system is applicable to all specula and dilators, together with their related explorers and fixed cutting instruments, for whatever part designed — the male or female urethra, the rectum, vagina, cervix uteri, œsophagus, Eustachian tube, or the lachrymal duct.

In designating sizes and recording data by the perimetric dimension system, millimeters will naturally be used for the smaller instruments and passages, while for the larger, as rectal and vaginal, centimeters should be employed. The changed form of expression will then be, for example, 20 M. M. instead of No. 20, French catheter — a gain in explicitness with no loss of brevity; and in place of Sim's No. 1 vaginal dilator, as at present, its equivalent, 10 C. M.; or, 8 C. M. as the proper substitute for No. 10 of English rectal bougies; or, again, 30 M. M. as closely approximating the dimensions of No. 18 of the American scale.

A comprehensive plan of unification is thus afforded, based upon the best known standard; for, whatever may be the faults of the metric system for general mechanical purposes, it is perfect for surgical uses. Neither can objection be raised to it in this case, on the ground of infraction of established routine, as is done in regard to its introduction into medicine and pharmacy, for in surgery there is no generally accepted standard to be displaced. In fact, except in the case of the urethral instruments before mentioned, there has been no attempt to indicate actual dimensions of any kind in the numbering of surgical instruments; while the sizes of nearly all appliances in use are purely arbitrary, if not in many instances simply the result of accident.

While the proposed system of measurement is fixed and definite, it yet allows entire freedom for individual choice, on the part of the surgeon in the gradation of the sizes of instruments, both as regards their number and their relative dimensions. It includes and utilizes all scales by giving them a common nomenclature; being especially in accord with the French urethral scale, however, for in this, though it is limited to certain fixed gradations, nominal number and actual size expressed in metric terms correspond.*

The importance of measurement by circumference or *perimeter*, instead of by diameter, is not to be overlooked, inasmuch as many instruments are irregular in outline, and therefore not susceptible of measurement by the latter method.

The adaptable metric gauge supplies a ready means for rendering the foregoing plan practicable, and thus securing the highest degree of definiteness and accuracy for purposes of record, comparison, and operative procedure. In illustration: During several years I have made somewhat frequent use of Otis's dilating urethrotome in obstinate and irritable stricture, and, though using at different times the best procurable makes of that admirable instrument, have found that a ready means of verifying or correcting its index was needed. One now in use, and an otherwise faultless piece of mechanism, being accurately measured over the knife in place, shows an excess of size over that registered of 4 M. M. An error like this, not recognized and provided against in an operation of such delicacy and gravity as that of Otis's for internal urethrotomy — in which the only hope of success depends upon strict accuracy and correspondence of measurements — may at any moment be the cause of serious mischief, or even of fatal results. Again, Ellinger's dilator for the cervix uteri has a seemingly perfect parallel motion; but when measured by the gauge shows conicity of 12 M. M., which is increased to 2 C. M., or more by pressure near the points when its sides are separated. Its failure to be retained when in use is thus accounted for. Or, an instance mentioned by a friend, a steel sound which had been looked upon as standard 32 French, proved upon measurement to be fully 39.5 M. M.— an enormous error.

The gauge is a simple appliance, mechanically similar to the glovers' measure, and consists of a narrow, flexible, measuring tape, graduated in centimeters and millimeters (Fig. 1), to which is attached a hand-piece, having a mortise for the passage of the

* The American scale, with its distinctive gradations, may be virtually reproduced by the same method, by making successive advances in size of 1.5 M. M. in circumference, as 1, 2.5, 4 5.5, 7 M. M., etc.

tape. A sliding loop is thus formed (Fig. 2), within which instruments to be measured are placed.

Fig. 2.

The two ends of the gauge being drawn upon in opposite directions so as snugly to embrace the enclosed object, the dimensions of its circumference if cylindrical, or its perimeter if of irregular outline, are indicated by arrows placed opposite the point of beginning of the scale.

The material found best adapted for its construction is an extra-heavy bank-note or bond paper, the handle being stiffened with card-board. This paper is very flexible, strong, and durable, is readily printed in fine, but legible, divisions, bears all ordinary use without stretching or breaking, and is not perceptibly affected by atmospheric changes. In practice it answers well for all purposes, including measurements involving delicate cutting edges.

Accurate to a fraction of a millimeter, the gauge becomes an instrument of precision, adapted to ascertaining the perimeters of a great variety of forms, and to expressing their values in uniform terms. It has the special advantage of utilizing old appliances, for by it their equivalence under the general system may be at once determined.

Contrasted with the ordinary gauge-plate, the adaptable gauge will be seen to be possessed of important advantages. The former is capable of measuring cylindrical forms only, and as made, is often inaccurate and always very limited in its range of sizes. The adaptable metric gauge, on the contrary, beside being accurate, is practically unlimited in capacity; measures cylinders perfectly, and is equally well adapted to the measurement of instruments of irregular outlines, as urethrotomes, metrotomes, separable dilators, divulsers, folding specula, and the like. While the gauge-plate is difficult of verification, the correctness of the adaptable gauge may be instantly tested by comparison with any standard metric rule.

109 SOUTH EIGHTH STREET,
PHILADELPHIA, June 10, 1879.

CHARLES H. THOMAS, M. D.,

*Dear Doctor:* We are now using your adaptable metric gauge as a correct guide in the manufacture of urethral and dilating instruments. It has been particularly serviceable in enabling us to bring the measurement of bougies, catheters, and all urethral-cutting instruments to the point of absolute accuracy.

Thanking you for bringing the gauge to our notice, we would state that we have made arrangements to produce them, and should be most happy to furnish members of the profession with your very useful appliance gratuitously.

Your views in regard to a universal scale are so evidently correct that we are prepared to conform to them.

We are, very respectfully, yours,
J. H. GEMRIG & SONS.

They will be also furnished, without cost, upon application to Messrs. George Tiemann & Co., New York City; D. W. Kolbé & Son, Philadelphia, Pa; Sharp & Smith, Chicago, Ills.; or the American Metric Bureau, Boston, Mass.—From *Philadelphia Medical Times.* Revised, with additions, by the author.

---

ORIGIN OF PLANTS.—Cabbage grew wild in Siberia; buckwheat originated in Siberia; celery originated in Germany; the potato is a native of Peru; the onion originated in Egypt; tobacco is a native of South America; millet was first discovered in India; the nettle is a native of Europe; the citron is a native of Asia; oats originated in North Africa; rye came originally from Siberia; parsley was first discovered in Sardinia; the parsnip is a native of Arabia; the sun-flower was brought from Peru; spinach was first cultivated in Arabia; the pear and apple are from Europe; the horse chestnut is a native of Thibet; the quince came from Island of Crete; the radish is a native of China and Japan; the pear is supposed to be of Egyptian origin; the horse radish came from the south of Europe.

## Figures and Tables

**Fig. 1. f1:**
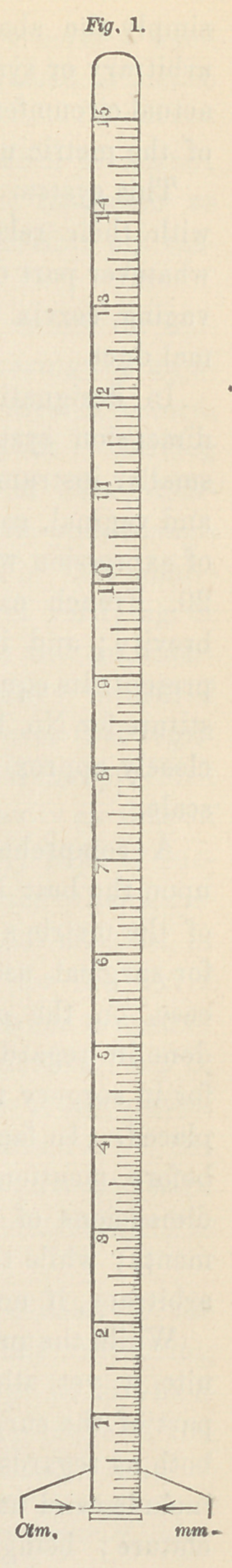


**Fig. 2. f2:**